# Triceps surae muscle hypertrophy is greater after standing *versus* seated calf-raise training

**DOI:** 10.3389/fphys.2023.1272106

**Published:** 2023-12-13

**Authors:** Momoka Kinoshita, Sumiaki Maeo, Yuuto Kobayashi, Yuuri Eihara, Munetaka Ono, Mauto Sato, Takashi Sugiyama, Hiroaki Kanehisa, Tadao Isaka

**Affiliations:** ^1^ Faculty of Sport and Health Science, Ritsumeikan University, Kusatsu, Japan; ^2^ Institute of Advanced Research for Sport and Health Science, Ritsumeikan University, Kusatsu, Japan; ^3^ Department of Physical Education, National Institute of Fitness and Sports in Kanoya, Kanoya, Japan

**Keywords:** bi- and monoarticular muscles, muscle length, muscle volume, resistance training, selective hypertrophy

## Abstract

**Background:** The triceps surae muscle plays important roles in fundamental human movements. However, this muscle is relatively unresponsive to resistance training (difficult to hypertrophy) but prone to atrophy with inactivity compared with other muscles. Thus, identifying an effective training modality for the triceps surae is warranted. This study compared triceps surae muscle hypertrophy after standing/knee-extended *versus* seated/knee-flexed plantarflexion (calf-raise) training, where the gastrocnemius is lengthened and shortened, respectively.

**Methods:** Fourteen untrained adults conducted calf-raise training with one leg in a standing/knee-extended position and the other leg in a seated/knee 90°-flexed position at 70% of one-repetition maximum. Each leg performed 10 repetitions/set, 5 sets/session, 2 sessions/week for 12 weeks. Before and after the intervention, magnetic resonance imaging scans were obtained to assess muscle volume of each and the whole triceps surae.

**Results**: Muscle volume significantly increased in all three muscles and the whole triceps surae for both legs (*p* ≤ 0.031), except for the gastrocnemius muscles of the seated condition leg (*p* = 0.147–0.508). The changes in muscle volume were significantly greater for the standing than seated condition leg in the lateral gastrocnemius (12.4% vs. 1.7%), medial gastrocnemius (9.2% vs. 0.6%), and whole triceps surae (5.6% vs. 2.1%) (*p* ≤ 0.011), but similar between legs in the soleus (2.1% vs. 2.9%, *p* = 0.410).

**Conclusion:** Standing calf-raise was by far more effective, therefore recommended, than seated calf-raise for inducing muscle hypertrophy of the gastrocnemius and consequently the whole triceps surae. This result and similar between-condition hypertrophy in the soleus collectively suggest that training at long muscle lengths promotes muscle hypertrophy.

## Introduction

The triceps surae muscle is the main plantarflexor and plays important roles in fundamental human movements such as walking (Ericson et al., 1986), running (Willer et al., 2021) and jumping (Hof et al., 2002). Since the triceps surae also functions as a stabilizer of our body (Honeine et al., 2013), the fall risk is related to the size/strength of the triceps surae (Allison et al., 2018; Epro et al., 2018). However, this muscle is relatively unresponsive to resistance training (difficult to hypertrophy) (Weiss et al., 1988; Ferri et al., 2003) but prone to atrophy with inactivity (Akima et al., 2001; Alkner and Tesch, 2004) compared with other muscles. Thus, enhancing our knowledge of effective training modalities for the triceps surae will be highly useful in sports and clinical settings, and will consequently benefit a wide range of population.

**FIGURE 1:**
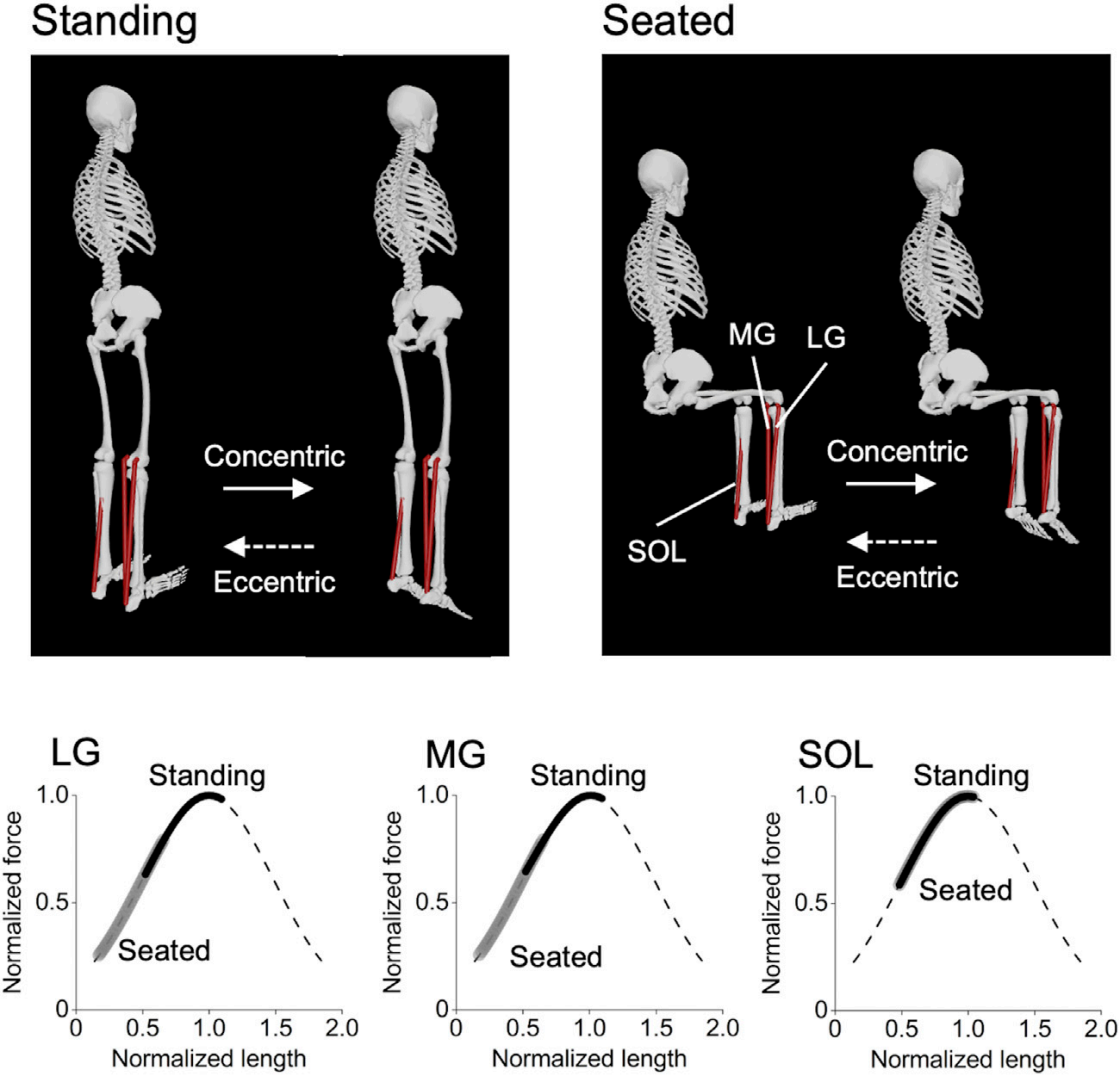
Postures of the standing and seated calf-raise exercises and operating ranges of each triceps surae muscle on the normalized force–length curve during the exercises. These were obtained using the OpenSim Gait 2392 model (Delp et al., 2007), with the knee joint 0° and 90° for the standing and seated conditions, respectively, and the ankle joint angle ranging from 20° dorsiflexed to 30° plantarflexed positions for both conditions. It can be clearly seen that the lateral and medial gastrocnemius (LG and MG) operate at longer muscle lengths in the standing than seated condition, while there is no difference between the conditions in the soleus (SOL).

The triceps surae consists of the lateral gastrocnemius (LG), medial gastrocnemius (MG), and soleus (SOL). The LG and MG are biarticular muscles crossing the knee joint, and are lengthened more in a knee-extended than knee-flexed position (Delp et al., 2007; Wakahara et al., 2007) (Figure 1). Recent studies (Maeo et al., 2021; Maeo et al., 2023) have shown that, by manipulating an angle of one of the two joints the associated biarticular muscles cross, training-induced muscle hypertrophy is greater after training at long than short muscle lengths. For example, hamstring muscle hypertrophy was greater after seated (hip-flexed) than prone (hip-extended) leg curl training (Maeo et al., 2021). In addition, triceps brachii muscle hypertrophy was greater after overhead (shoulder-flexed) than push down (shoulder-extended) cable elbow extension training (Maeo et al., 2023). Based on these, triceps surae muscle hypertrophy may be greater after standing than seated calf-raise training. Both standing and seated calf-raise are common exercises to train the triceps surae, and have been often implemented alone (Carson et al., 2010; Gavanda et al., 2020; Kudo et al., 2020) or in combination (Schoenfeld et al., 2020; Kataoka et al., 2022; Van Every et al., 2022) in previous studies. However, no study has compared their hypertrophic effects through training interventions. Thus, clarifying their comparative hypertrophic effects will provide simple yet highly practical information for developing evidence-based training programs for the triceps surae, and consequently other muscles as well.

The purpose of this study was to compare triceps surae muscle hypertrophy after standing (more stretched) *versus* seated (less stretched) calf-raise training. To this end, we designed a 12-week training intervention study using a within-person comparison model (Maeo et al., 2021; Maeo et al., 2023). This model is powerful when comparing hypertrophic effects of two training modalities by largely reducing the potential influence of between-person variability in gene expression and other factors (e.g., habitual diet and activity/sleep) on the results obtained (MacInnis et al., 2017). This study solely focused on muscle hypertrophy because training effects on muscle strength and other functional performances may be confounded (e.g., by the cross-education effect) in this comparison model (MacInnis et al., 2017). We hypothesized that triceps surae muscle hypertrophy would be greater after standing than seated calf-raise training.

## Methods

### Participants and overview

Fourteen untrained healthy adults (7 females/males, age: 23.3 ± 2.4 years, height: 167.3 ± 7.9 cm, body mass: 56.6 ± 19.7 kg) participated in this study, which was approved by the Ethics Committee of Ritsumeikan University (BKC-LSMH-2019-019). We did not control female participants’ menstrual cycle. This was because of 1) no apparent effect of its cycle on training-induced muscle hypertrophy (Colenso-Semple et al., 2023) and 2) our within-person comparison approach as explained above (MacInnis et al., 2017). Written informed consent was obtained from each participant. They performed calf-raise training with one leg in a standing/knee-extended position (Standing-Leg) and the other leg in a seated/knee 90°-flexed position (Seated-Leg). Before and after intervention of 12 weeks, magnetic resonance imaging (MRI) scans were obtained to assess muscle volume, the gold-standard muscle size measure (Cruz-Jentoft et al., 2010), of each and the whole triceps surae (Whole-TS).

### Training program

**FIGURE 2:**
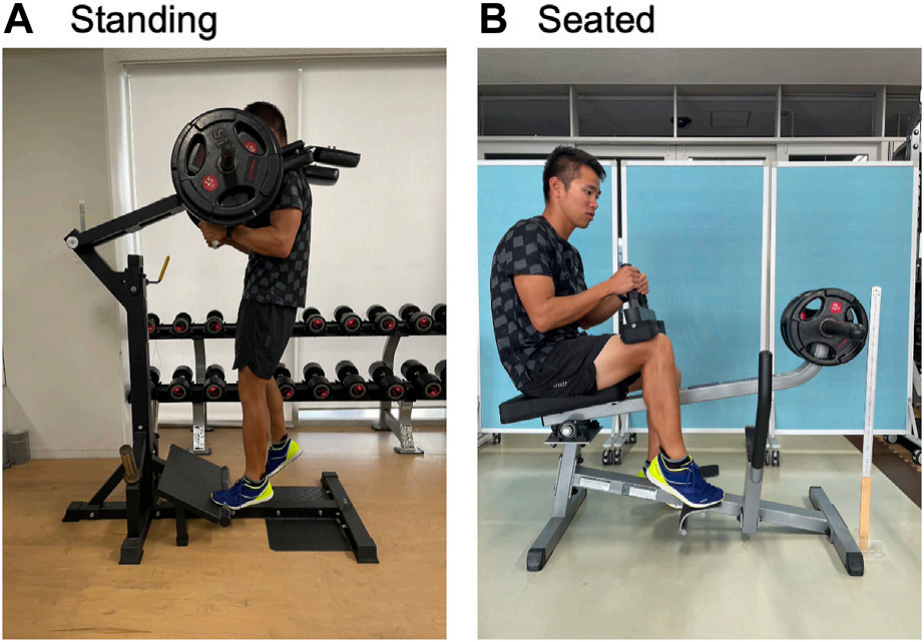
Pictures of standing **(A)** and seated **(B)** calf-raise training, targeting the left and right leg, respectively.

Each leg was assigned to Standing-leg or Seated-leg with the dominant and non-dominant legs counterbalanced, and trained unilaterally using standing (IMH703, Coming Health Tech, Qingdao, China) and seated (GSCR349, Bodysolid, Forest Park, Illinois, United States) calf-raise machines. Participants were instructed to place/keep their foot in a neutral position (Figure 2) to avoid potential confounding influence of foot positioning (Nunes et al., 2020). Following warm-up repetitions at 50% (x10) and 80% (x5) of each session’s training load (detailed below), participants performed the standing or seated calf-raise 10 repetitions per set for 5 sets, taking 2 s for each of the concentric/lifting and eccentric/lowering phases with the guide of a metronome (60 bpm). This duration/tempo was chosen so that the exercises were performed in a controlled manner (Maeo et al., 2021; Maeo et al., 2023). Two-min rest intervals were taken in between sets. After training one leg (5 sets), the other leg was trained, the order of which was switched every session. Training was conducted twice per week on non-consecutive days for 12 weeks. Training set was gradually increased from 3, 4, and 5 sets at the first, second, and third sessions, respectively, using a consistent load of 70% of one-repetition maximum (1RM) measured pre-training. At least one examiner always supervised the training sessions, provided verbal encouragement, and corrected the joint positions and/or movement speed when necessary. The examiner also assisted/spotted the participants when they could no longer repeat the repetitions up to 10 in each set. If the participants could complete all the prescribed protocol at the third session and thereafter without the examiner’s assistance, +5% of 1RM was added at the subsequent sessions, with the load of the unloaded machines considered for the load calculation. The above training protocol was based on the ACSM guidelines (ACSM, 2009; Garber et al., 2011) and our previous studies (Maeo et al., 2021; Maeo et al., 2023) on other muscles that found typical/greater hypertrophy compared to the values reported in their relevant literature.

### MRI

Longitudinal relaxation time-weighted cross-sectional MRI scans were obtained for each leg using body array and spine coils (Body 18 and CP Spine Array Coil, Siemens Healthineers, Erlangen, Germany) with the following basic parameters: field of view, 200 × 200 mm; slice thickness and gap, 5 mm; voxel size, 0.39 × 0.39 × 5 mm; TR, 700 ms; TE, 10 ms, number of slices, 20 × 2 blocks. Participants lay supine with their legs extended and muscles relaxed in a 3-T magnet bore (MAGNETOM Skyra, Siemens Healthineers, Germany).

Images were analyzed by using image analysis software (Horos, v3.3.6, Horos Project), with the MRI data anonymized and investigators blinded to the training conditions. Anatomical cross-sectional areas (ACSAs) of the individual triceps surae were manually outlined in every other image from the most proximal to the most distal image in which the muscle was visible. ACSAs for the skipped images and gaps were estimated based on linear interpolation between the images in which ACSAs were outlined. The volume of individual muscle was determined by summing all ACSAs for that muscle multiplied by the slice thickness. The Whole-TS volume was calculated by summing the volumes of the individual muscles. Intra-rater repeatability for measuring muscle volume of each muscle was assessed on eight legs. The coefficient of variation (CV) was 9.8%, 2.2%, and 1.8% for the LG, MG, and SOL, respectively.

### Statistical analysis

Descriptive data are presented as mean ± SD. All data were analyzed using SPSS software (version 28.0, IBM, Armonk, New York, United States). Statistical significance was set at *p* < 0.05. Males and females were analyzed together because training-induced muscle hypertrophy is known to be similar between sexes (Roberts et al., 2020) and separate analyses largely reduce statistical power (MacInnis et al., 2017). We ensured independence of observations (each participant was only counted as one observation in each condition). Normality was confirmed for all dataset by the Shapiro-Wilk test. A two-way repeated-measures ANOVA (time×leg) was used for a comparison of muscle volume for each muscle and the Whole-TS. Homoscedasticity and sphericity was checked by scatterplots and Mauchly’s test in ANOVA, respectively, and *p* values were modified with Greenhouse–Geisser correction when necessary. When a significant interaction was found, then *post hoc* tests were conducted. Specifically, a paired *t*-test was used to test the difference between the pre- and post-training values within each leg. Additionally, absolute change values were also calculated and compared between legs by using an unpaired *t*-test. Effect sizes of between-condition differences were calculated as Cohen’s *d* values based on absolute change values, and were interpreted as trivial <0.2; small 0.2–0.49; moderate 0.5-0.79; and large ≥0.8 (Cohen, 1988). Finally, their bootstrap 95% confidence interval (5,000 samples, bias-corrected and accelerated) was assessed for each leg by using estimation statistics (Ho et al., 2019) to improve statistical inference.

## Results

**FIGURE 3:**
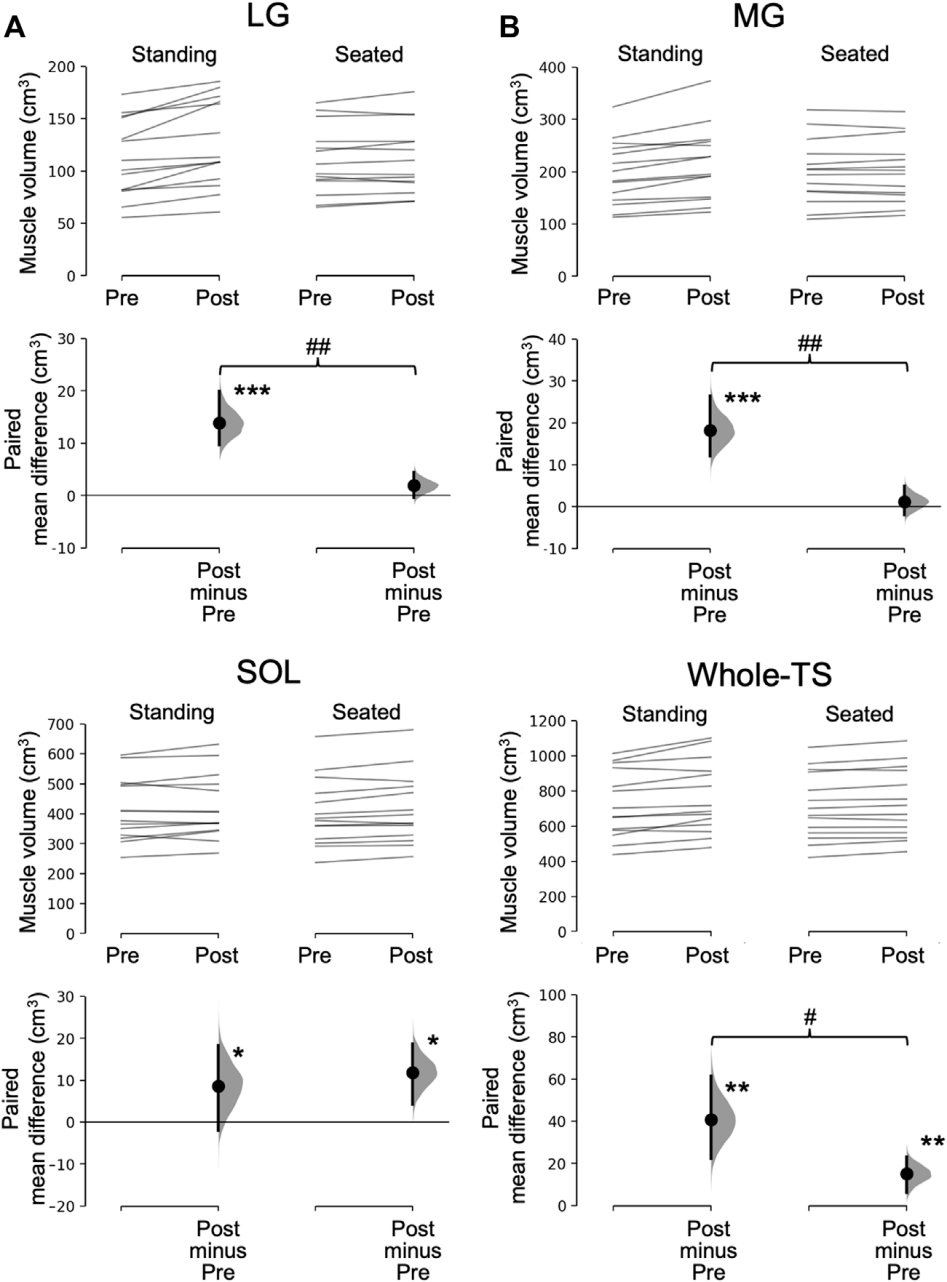
Muscle volume before and after the training and its change. In each subfigure/muscle(s), the raw data is plotted on the upper axes for the standing **(A)** and seated **(B)** conditions; each paired set of observations at Pre and Post is connected by a line. On the lower axes, each paired mean difference is plotted as a bootstrap sampling distribution. Mean differences are depicted as dots with horizontal dashed lines; 95% confidence intervals are indicated by the ends of the vertical error bars. ***p < 0.001, **p < 0.01 and *p < 0.05 difference between times (pre vs. post). ##*p* < 0.01 and #*p* < 0.05 difference between conditions (legs). LG, lateral gastrocnemius; MG, medial gastrocnemius; SOL, soleus; Whole-TS, whole triceps surae.

**FIGURE 4:**
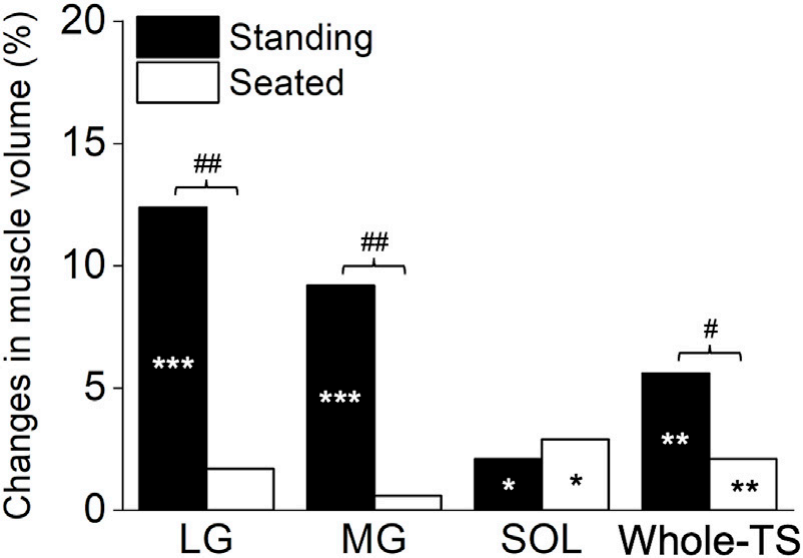
The summary in percentage change based on the mean changes for each muscle and the whole triceps surae. ***p < 0.001, **p < 0.01 and *p < 0.05 difference between times (pre vs. post). ##*p* < 0.01 and #*p* < 0.05 difference between conditions (legs). LG, lateral gastrocnemius; MG, medial gastrocnemius; SOL, soleus; Whole-TS, whole triceps surae.

Significant time×leg interactions were found in the muscle volume of the LG (*p* = 0.001), MG (*p* = 0.002), and Whole-TS (*p* = 0.011), but not in the SOL (*p* = 0.411) which albeit had a main effect of time (*p* = 0.031). Paired t-tests within each leg for the LG, MG, and Whole-TS, as well as the main effect of time for the SOL, revealed that muscle volume significantly increased in all three muscles and the Whole-TS for both legs (*p* ≤ 0.031), except for the LG and MG of the Seated-Leg (*p* = 0.147–0.508) (Figure 3). The changes in muscle volume were significantly greater for the Standing-Leg than Seated-Leg in the LG (12.4% vs. 1.7%, *p* = 0.001, Cohen’s *d* = 1.53 [large]), MG (9.2% vs. 0.6%, *p* = 0.002, *d* = 1.58 [large]) and Whole-TS (5.6% vs. 2.1%, *p* = 0.011, *d* = 0.88 [large]), but similar between legs in the SOL (2.1% vs. 2.9%, *p* = 0.410, *d* = 0.2 [trivial]) (Figures 3, 4).

## Discussion

The main finding of this study was that muscle hypertrophy of the gastrocnemius, and consequently the Whole-TS, was significantly greater after standing than seated calf-raise training, indicating that training at long muscle lengths promotes muscle hypertrophy. Furthermore, no significant hypertrophy was found in the gastrocnemius after seated calf-raise training. This suggests that training at short muscle lengths could result in no/negligible hypertrophy of the otherwise trained/hypertrophied muscles.

To the authors’ knowledge, this is the first study to reveal changes in MRI-measured muscle volume for each and the Whole-TS after calf-raise training. Importantly, the results clearly showed that muscle hypertrophy was greater after standing than seated calf-raise training, indicating greater hypertrophic effects of training at long muscle lengths. While beyond the scope of this study, potential mechanisms for greater hypertrophy after training at long muscle lengths, examined using isometric or traditional (involving both concentric/eccentric) exercises, include greater muscle hypoxia/metabolic stress (Kooistra et al., 2008), IGF-1 expression (McMahon et al., 2014), and muscle damage (Allen et al., 2018). These alone or in combination could promote muscle hypertrophy/regeneration (Wackerhage et al., 2018). Additionally, other studies using electromyography (Price et al., 2003; Arampatzis et al., 2006; Wakahara et al., 2007), ultrasonography (Kassiano et al., 2023a), and functional MRI (Price et al., 2003) showed that the LG and/or MG are more activated when isometric or traditional plantarflexion/calf-raise exercise is performed in a knee-extended position. These would collectively explain why standing calf-raise training produced greater hypertrophy of the gastrocnemius and consequently the Whole-TS, compared to seated calf-raise training.

Interestingly, there were no significant changes in the gastrocnemius muscle volumes after seated calf-raise training. Among the studies that conducted training at short muscle lengths, some reported small yet significant hypertrophy (McMahon et al., 2014; Maeo et al., 2021; Maeo et al., 2023), but others found no significant changes in muscle size (Noorkoiv et al., 2014; Kassiano et al., 2023b). No significant hypertrophy in the previous two studies (Noorkoiv et al., 2014; Kassiano et al., 2023b) could be at least partly due to their relatively short training periods (6–8 weeks). However, this study adopted a training period of 12 weeks, and therefore our result is less likely attributed to the lack/shortness of training period. Rather, no significant hypertrophy in the previous studies (Noorkoiv et al., 2014; Kassiano et al., 2023b) and this study may be partly attributable to the fact that all studies targeted antigravity muscles of the legs (the triceps surae or quadriceps), which are known to be less prone to hypertrophy (Usui et al., 2016; Maeo et al., 2018) than other muscles (Kohiruimaki et al., 2019; Maeo et al., 2021; Maeo et al., 2023), likely due to being habitually activated in daily activities (Folland and Williams, 2007). Thus, while there are some differences among studies in the methodologies adopted, this study provides the first convincing evidence that training at short muscle lengths could lead to no/negligible hypertrophy even after 12 weeks of training, in other words could “minimize” muscle hypertrophy.

This study has some limitations. First, we did not set a control group. However, the CV of intra-rater repeatability for measuring muscle volume was 9.8%, 2.2%, and 1.8% for the LG, MG, and SOL, respectively. The first two values, especially for the MG, were smaller than the significant between-condition differences in the LG (12.4% vs. 1.7%) and MG (9.2% vs. 0.6%). Furthermore, the CV for the SOL was smaller than the significant hypertrophy of both conditions (2.1% vs. 2.9%). Given that we analyzed the data in a blinded/anonymized manner, the findings obtained here would be robust/unchanged even if we had a control group. The somewhat higher CV for the LG than the other two muscles may be due to its small size (Figure 3). Such information may be useful when selecting which muscle to analyze within the triceps surae (i.e., MG or SOL may be better) when any muscle can be analyzed depending on a research purpose. Additionally, no functional data is available in this study. As mentioned earlier, this study prioritized muscle size measurement/comparison. Therefore, we used the within-person comparison model, which may not be best suited for comparisons of functional performances (MacInnis et al., 2017). Because muscle strength and power are strongly related to muscle size (Kordi et al., 2020; Maden-Wilkinson et al., 2020; Balshaw et al., 2021), improvements in functional performances would have been also better for the standing vs. seated calf-raise training. Nevertheless, more studies are needed to directly investigate the effect of standing vs. seated calf-raise training on various functional performances.

## Conclusion

The degrees of muscle hypertrophy of the triceps surae were relatively small (∼2–6%), as previously reported, compared to other muscle groups (e.g., +9–20% in the hamstrings and triceps brachii, Maeo et al., 2021; Maeo et al., 2023) after 12 weeks of training. However, we found a clear difference in the hypertrophic responses after standing vs. seated calf-raise training. Specifically, standing calf-raise was by far more effective than seated calf-raise for inducing muscle hypertrophy of the gastrocnemius and consequently the whole triceps surae (Cohen’s *d* = 0.88–1.58 [large]). Considering the limited hypertrophic response of the triceps surae and its proneness to atrophy with inactivity, the finding of this study will be readily useful in sports and clinical settings in maximizing hypertrophy and minimizing atrophy of the triceps surae.

## Data Availability

The original contributions presented in the study are included in the article/Supplementary Material, further inquiries can be directed to the corresponding author.
